# Rejection of Care in Hospitalized Persons Living With Dementia: The Impact of Nurse Communication

**DOI:** 10.1093/geroni/igab046.2234

**Published:** 2021-12-17

**Authors:** Clarissa Shaw, Caitlin Ward, Jean Gordon, Kristine Williams, Keela Herr

**Affiliations:** 1 University of Iowa College of Nursing, Iowa City, Iowa, United States; 2 University of Iowa, Iowa City, Iowa, United States; 3 University of Kansas School of Nursing, Kansas City, Kansas, United States

## Abstract

Rejection of care (RoC) by persons living with dementia (PLWD) has yet to be measured in the hospital setting. Elderspeak communication (i.e., baby talk or infantilization) is an established antecedent to RoC in nursing home dementia care. The purpose of this study was to determine the impact of elderspeak communication by nursing staff on RoC by hospitalized PLWD. Eighty-eight care encounters between 16 PLWD and 53 nursing staff were observed for RoC using the Resistiveness to Care scale in one Midwestern hospital. Audio-recordings of the care encounters were transcribed verbatim and coded for semantic, pragmatic, and prosodic features of elderspeak. Over one-quarter (28.7%) of the duration of nursing staff speech towards PLWD constituted elderspeak and nearly all (96.6%) of the 88 care encounters included some elderspeak. Almost half of the observations (48.9%) included RoC behaviors by PLWD. Rejection of care was modeled as present or absent using a GEE method. Characteristics of the PLWD (e.g., pain, delirium) and the observation (e.g., environmental simulation) were evaluated as potential covariates. After adjusting for pain, length of stay, and gender, a 15-percentage point decrease in the proportion of elderspeak communication by nursing staff reduced the odds of RoC by 62% (OR=0.38, 95% CI=0.21-0.71, p=.002,) and a one unit decrease in pain reduced the odds of RoC by 63% (OR=0.37, 95% CI=0.22-0.63, p<.001). This study identified that pain and elderspeak are two modifiable factors of RoC. Person-centered interventions are needed that address communication practices and approaches to pain management for hospitalized PLWD.

